# Clinical Proofs of Distinct Motory and Percepient Nerves

**Published:** 1834

**Authors:** 


### VIII.—Clinical proofs of distinct Nerves for Motion and Sensation.

We cannot doubt, that the practice of medicine, might furnish to the acute observer, many striking evidences of the truth of Sir Charles Bell's discovery, that there are distinct sets of nerves for motion and sensation. Without having been as attentive to this matter as we ought, we have within the last few years met with several cases of paralysis that were instructive on this point.

1. Being called to an elderly woman, suddenly taken down with hemiplegia, which proved fatal, we proceeded to bleed her; and performed the operation in the arm of the affected side, as happening to be most convenient. On making the puncture, although previously unaware of what was impending she screamed out from the pain, and threw her other arm about with violence; but that in which the puncture was made, remained as motionless as if it had been dead.

2. About the same period we saw, for a single time only, a young woman on board a steam boat, who had very little sensibility in one side, while its power of motion was nearly unimpaired. She appeared to be recovering; but we never had an opportunity of learning the termination of the case. The disease had come upon her suddenly.

3. Very lately, a gentleman from the south, presented himself to us with a paralysis of one side of his face, of six or eight weeks standing. The forehead of that side, as is usual in such cases, was smooth, the eyelids could not be closed without a great effort, and the angle of the mouth hung a a little, and was drawn forward by the superior action of the antagonizing muscles of the other side. Notwithstanding all this loss of power in the motory nerves of that side of the face, its sensibilities were perfect, both in relation to heat and cold, and to mechanical irritation of every kind.

We would earnestly recommend to our readers, to be diligent in their observation on cases of paralysis, with a view to this subject.

